# High-Flow Nasal Cannula for COVID-19 Patients: A Multicenter Retrospective Study in China

**DOI:** 10.3389/fmolb.2021.639100

**Published:** 2021-04-13

**Authors:** Jun Duan, Jia Zeng, Puyu Deng, Zhong Ni, Rongli Lu, Wenxi Xia, Guoqiang Jing, Xiaoping Su, Stephan Ehrmann, Wei Zhang, Jie Li

**Affiliations:** ^1^ Department of Respiratory and Critical Care Medicine, First Affiliated Hospital of Chongqing Medical University, Chongqing, China; ^2^ Department of Aviation Disease, Naval Medical Center of PLA, Second Military Medical University, Shanghai, China; ^3^ Hubei Maternal and Child Health Hospital, Wuhan, China; ^4^ Department of Emergency and Critical Care Medicine, Shanghai General Hospital, Shanghai Jiao Tong University School of Medicine, Shanghai, China; ^5^ Department of Respiratory and Critical Care Medicine, West China Hospital, Sichuan University, Chengdu, China; ^6^ Department of Pulmonary and Critical Care Medicine, Xiangya Hospital, Central South University, Changsha, China; ^7^ Department of Critical Care Medicine, West China Hospital, Sichuan University, Chengdu, China; ^8^ Department of Pulmonary and Critical Care Medicine, Binzhou Medical University Hospital, Binzhou Medical University, Binzhou, China; ^9^ School of Basic Medicine, Wenzhou Medical University, Wenzhou Tea Mountain Higher Education Park, Wenzhou, China; ^10^ CHRU Tours, Médecine Intensive Réanimation, CIC INSERM 1415, CRICS-TriggerSep Network, Tours France, and INSERM, Centre D’étude des Pathologies Respiratoires, Université de Tours, Tours, France; ^11^ Department of Respiratory and Critical Care Medicine, First Affiliated Hospital, Second Military Medical University, Shanghai, China; ^12^ Department of Cardiopulmonary Sciences, Division of Respiratory Care, Rush University Medical Center, Chicago, IL, United States

**Keywords:** coronavirus, high-flow nasal cannula, ROX index, risk factor, delay intubation

## Abstract

**Background:** High-flow nasal cannula (HFNC) may help avoid intubation of hypoxemic patients suffering from COVID-19; however, it may also contribute to delaying intubation, which may increase mortality. Here, we aimed to identify the predictors of HFNC failure among patients with COVID-19.

**Methods:** We performed a multicenter retrospective study in China from January 15 to March 31, 2020. Two centers in Wuhan (resource-limited centers) enrolled 32 patients, and four centers outside Wuhan enrolled 34 cases. HFNC failure was defined as the requirement of escalation therapy (NIV or intubation). The ROX index (the ratio of SpO_2_/FiO_2_ to the respiratory rate) was calculated.

**Results:** Among the 66 patients, 29 (44%) cases experienced HFNC failure. The ROX index was much lower in failing patients than in successful ones after 1, 2, 4, 8, 12, and 24 h of HFNC. The ROX index was independently associated with HFNC failure (OR = 0.65; 95% CI: 0.45–0.94) among the variables collected before and 1 h after HFNC. To predict HFNC failure tested by ROX index, the AUC was between 0.73 and 0.79 for the time points of measurement 1–24 h after HFNC initiation. The HFNC failure rate was not different between patients in and outside Wuhan (41% vs. 47%, *p* = 0.63). However, the time from HFNC initiation to intubation was longer in Wuhan than that outside Wuhan (median 63 vs. 22 h, *p* = 0.02). Four patients in Wuhan underwent intubation due to cardiac arrest; in contrast, none of the patients outside Wuhan received intubation (13 vs. 0%, *p* = 0.05). The mortality was higher in Wuhan than that out of Wuhan, but the difference did not reach statistical significance (31 vs. 12%, *p* = 0.07).

**Conclusion:** The ROX index can be used to predict HFNC failure among COVID-19 patients to avoid delayed intubation, which may occur in the resource-limited area.

## Introduction

As of January 17, 2021, more than ninety million cases were confirmed with 2019 novel coronavirus disease (COVID-19) worldwide, with a fatality rate of approximately 2% (WHO Coronavirus Disease Dashboard, 2021). Nearly 20% of patients experienced hypoxemia, which was the primary reason for hospitalization (Wu and McGoogan, 2020). Oxygen therapy is the primary treatment for those hypoxemic patients. In recent years, high-flow nasal cannula (HFNC) has been proven to improve oxygenation and ultimately reduce intubation rates for hypoxemic respiratory failure patients of various etiologies (Li et al., 2020a). HFNC provides gas flow higher than the patient's inspiratory flow demand, which enables the delivery of a constant fraction of inspired oxygen (FiO_2_) without dilution by room air. It also washes out the dead space and provides, to some extent, positive expiratory pressure (Nishimura, 2016).

Two retrospective studies with a small sample size from China reported that HFNC could improve oxygenation for COVID-19 patients, particularly among patients with PaO_2_/FiO_2_ > 200 mmHg (Geng et al., 2020; Wang et al., 2020). Among moderate-to-severe hypoxemic patients treated with HFNC, 36% of them did not require therapy escalation, such as intubation or noninvasive ventilation (NIV) (Wang et al., 2020). In Wuhan, China, 63.5% of ICU patients suffering from COVID-19 used HFNC (Yang et al., 2020). In Jiangsu, China, HFNC became the standard of care for hypoxemic COVID-19 patients (Sun et al., 2020). In the Seattle region, United States, 42% of critically ill patients received HFNC (Bhatraju et al., 2020). As the risk of virus transmission associated with HFNC is relatively low (Hui et al., 2019; Li et al., 2020b), current Surviving Sepsis Campaign COVID-19 subcommittee guidelines recommend using HFNC in hypoxemic patients with COVID-19 (Alhazzani et al., 2020). However, delayed intubation after HFNC failure is associated with increased mortality (Kang et al., 2015). Therefore, early identification of HFNC failure is essential, particularly in a resource-limited area, where the number of life-saving devices, such as ventilators, is limited; the use of those devices should be prioritized; early decision on the distribution of ventilators, instead of using a ventilator at the last minute, to patients with high possibility of HFNC failure might help reduce mortality (Kang et al., 2015).

The ROX index, the ratio of pulse oximetry (SpO_2_)/FiO_2_ to the respiratory rate, has been shown to effectively predict HFNC failure in patients with hypoxemia caused by bacterial pneumonia (Roca et al., 2016). However, its value for predicting HFNC failure in COVID-19 patients remains unknown. Albeit not fully elucidated so far, the pathophysiology of COVID-19–associated hypoxemia may differ from that of other diseases, such as bacterial pneumonia (Gattinoni et al., 2020a; Gattinoni et al., 2020b; Ziehr et al., 2020). In addition, the place where the device, such as the invasive ventilator, was unavailable when the patient required intubation was considered as a resource-limited area. Delayed intubation may occur in this area. As such, we aimed to identify the risk factors associated with HFNC failure in COVID-19 patients, and further explore the relationship between HFNC therapy and delayed intubation in a resource-limited area compared to a normal setting.

## Methods

A retrospective study was conducted in six Chinese hospitals, after approval by the institutional review board [approval No. FYGG(L)-2020–017], in the central institution (Guanggu, Wuhan). Adult patients with a laboratory-confirmed diagnosis of COVID-19 and treated by HFNC from January 15 to March 31, 2020 were enrolled. Exclusion criteria included 1) use of HFNC as palliative care and 2) use of HFNC for less than 30 min. Patients were identified by the medical record system in each hospital.

HFNC (Fisher & Paykel, Auckland, New Zealand; OH-70B/70C, Micomme Medical Technology, Hunan, China; and HiFent TM, Respircae Medical, Liaoning, China) was implemented according to the current consensus and experts’ suggestions (Respiratory and Critical Care Medicine Group of Chinese Thoracic Society, 2019; Critical care committee of Chinese Association of Chest Physician, 2020; Yuan et al., 2020). Flow and FiO_2_ were adjusted to maintain SpO_2_ above 93% and the respiratory rate below 30 breaths/min, while favoring patients’ tolerance. Withdrawal of HFNC was considered if FiO_2_ was less than 0.4. In case of respiratory failure worsening, escalation therapy consisting of NIV or intubation was initiated based on the attending physicians’ decision.

Patients’ demographic data, including age, gender, preexisting chronic diseases such as hypertension, diabetes mellitus, chronic pulmonary diseases, coronary artery disease, and cerebral infarction, and admission comorbidities, were collected. Chronic pulmonary disease included asthma, COPD, and bronchiectasis. Laboratory tests including white blood cell counts, cluster of differentiation 4 (CD4), lymphocyte counts, procalcitonin, IL-6, C-reactive protein, lactate dehydrogenase, lactic acid, and arterial blood gas analysis were also recorded, if available. The data of HFNC utilization including flow and F_i_O_2_ settings, patients’ changes in vital signs, and SpO_2_ at 1, 2, 4, 8, 12, and 24 h of HFNC were extracted from patients’ medical records. At the same time, the ROX index was calculated (Roca et al., 2016).

All the patients were followed up until discharge or death in the hospital. Data on HFNC duration, use of NIV as rescue therapy, intubation, survival, and length of stay in the ICU and hospital were collected. HFNC failure was defined as the requirement of escalation therapy (NIV or intubation) (Geng et al., 2020); HFNC failure in 28 days was recorded.

Among the six centers, two were in Wuhan and four were out of Wuhan. As many COVID-19 patients crowded into hospitals in Wuhan within a short period, the healthcare workers were overwhelmed and a severe shortage of medical devices occurred. Compared to the hospitals outside Wuhan, the resources in Wuhan were relatively inadequate. Thus, we defined the two centers in Wuhan as resource-limited areas, which probably impacted intubation decisions in patients who underwent HFNC.

### Statistical Analysis

Normally distributed continuous variables were reported as mean and standard deviation, and non-normally distributed continuous variables were reported as median and interquartile range (IQR). Differences between the groups of HFNC success and failure were analyzed using the Student’s *t*-test or Mann–Whitney *U* test when appropriate. Categorical variables were reported as number and percentage, and differences between groups were analyzed with using chi-square test or Fisher’s exact test when appropriate.

The area under the curve (AUC) of receiver operating characteristics was calculated to identify the predictive power of HFNC failure. The optimal cutoff value was determined at the maximal Youden index (Youden, 1950). Variables with a *p* value less than 0.1 in the univariate analysis were entered in a stepwise multivariate logistic regression analysis to identify independent risk factors associated with HFNC failure. As the respiratory rate, SpO_2_, and PaO_2_/FiO_2_ were collinear with the ROX index, they were not included in the regression analysis. A *p* value less than 0.05 was considered to be significant.

## Results

### Data Collected From Hospital Admission to Termination of High-Flow Nasal Cannula

**FIGURE 1:**
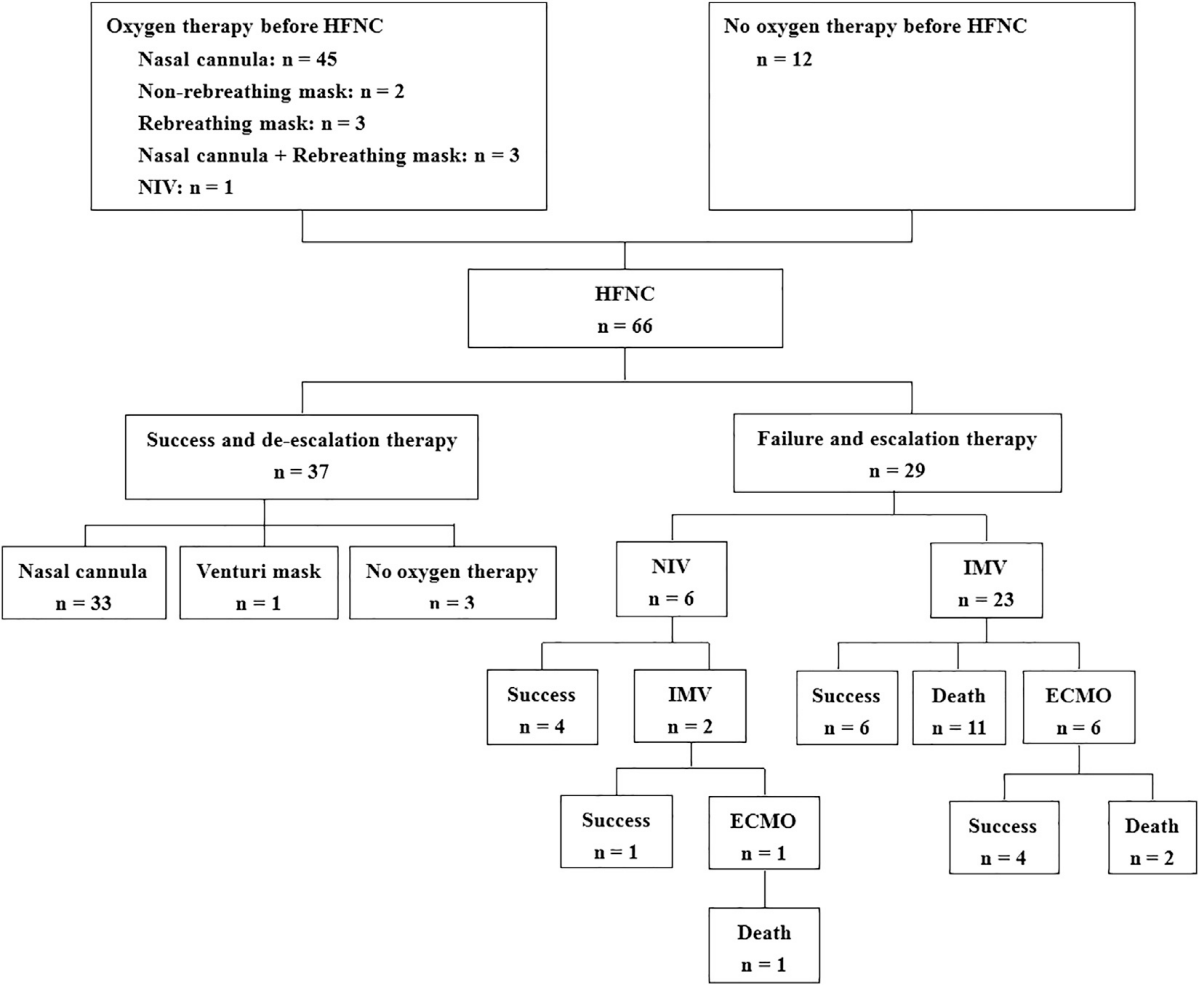
Flowchart of the enrolled patients. HFNC, high-flow nasal cannula; NIV, noninvasive ventilation; IMV, invasive mechanical ventilation; ECMO, extracorporeal membranous oxygenation.

We enrolled 66 patients with COVID-19 (32 in Wuhan and 34 outside Wuhan) in this study (Figure 1). Of them, 29 (44%) patients experienced HFNC failure and required escalation therapy within 28 days. Univariate comparisons of patients with HFNC success and failure are presented in Table 1. HFNC success was associated with younger age, lack of chronic respiratory disease, lower illness severity measured by the sequential organ failure score (SOFA), better oxygenation, less inflammation (lower procalcitonin levels), and immune dysfunction (higher lymphocyte count). However, the PaO_2_/FiO_2_ and ROX index did not differ between the two groups at hospital admission and before the use of HFNC (Table 1; Supplementary Table S1).

**TABLE 1:** Baseline data collected before the use of HFNC.

	HFNC success (*N* = 37)	HFNC failure (*N* = 29)	*P* value
Age, years	63 ± 16	73 ± 14	0.01
Male, n (%)	14 (38)	11 (38)	＞0.99
Oxygen therapy before HFNC, n %	8 (22)	4 (14)	0.53
SOFA score	3.4 ± 2.1	4.5 ± 1.7	0.047
Underlying disease, n %			
Hypertension	21 (57)	19 (66)	0.61
Diabetes mellitus	13 (35)	6 (21)	0.28
Coronary heart disease	4 (11)	4 (14)	0.72
Cerebral infarction	4 (11)	4 (14)	0.72
Chronic respiratory disease	3 (8)	8 (28)	0.048
Hypoproteinemia	6 (16)	7 (24)	0.54
Anemia	5 (14)	4 (14)	＞0.99
Chronic renal dysfunction	2 (5)	3 (10)	0.65
Gastrointestinal bleeding	1 (3)	3 (10)	0.31
Airway secretions, n %			
None	20 (54)	12 (41)	0.33
Mild	16 (43)	17 (59)	0.32
Moderate to abundant	1 (3)	0 (0)	＞0.99
Laboratory tests			
White blood cell counts, × 10^9^/L	8.5 ± 4.6	8.6 ± 3.5	0.94
Lymphocyte counts, × 10^9^/L	1.12 ± 0.95	0.59 ± 0.30	0.02
PCT, ng/mL	0.10 (0.05–0.14)	0.42 (0.10–2.37)	＜0.01
IL-6	8 (1–76)	73 (24–192)	0.13
C-reactive protein, mg/L	65 ± 53	96 ± 67	0.08
LDH, U/L	365 ± 114	429 ± 144	0.18
CD4, counts/μL	335 ± 183	152 ± 113	0.06
pH	7.42 ± 0.06	7.42 ± 0.08	0.77
PaCO_2_, mmHg	42 ± 9	37 ± 9	0.06
PaO_2_/F_i_O_2_, mmHg	214 ± 110	168 ± 108	0.15
Lactate, mmol/L	2.6 ± 1.2	2.8 ± 1.5	0.57
Vital signs			
Heart rate, beats/min	90 ± 11	93 ± 21	0.50
Respiratory rate, breaths/min	24 ± 4	26 ± 7	0.17
Systolic blood pressure, mmHg	122 ± 18	132 ± 22	0.07
Diastolic blood pressure, mmHg	70 ± 9	73 ± 10	0.33
SpO_2_, %	94 (92–96)	89 (85–93)	＜0.01
ROX index	9.4 ± 3.1	8.4 ± 4.7	0.32

HFNC, high-flow nasal cannula; SOFA, Sequential Organ Failure Assessment; PCT, procalcitonin; LDH, lactate dehydrogenase; ROX, the ratio of SpO_2_/F_i_O_2_ to the respiratory rate.

HFNC failure was defined as the requirement of escalation therapy (noninvasive ventilation or intubation).

### Outcomes

**FIGURE 2:**
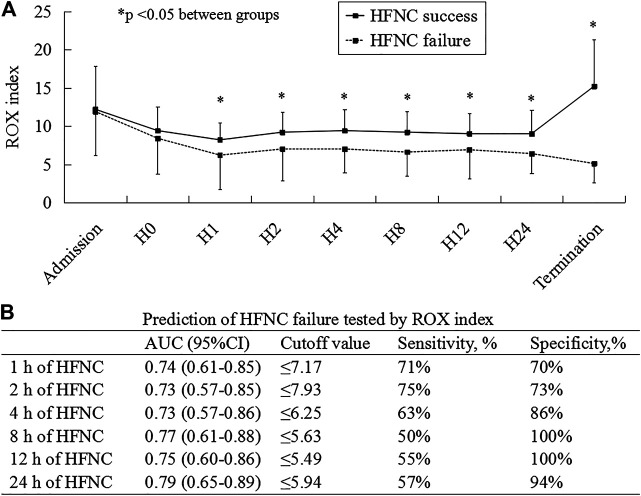
ROX index as a risk factor to predict HFNC failure. H0, H1, H2, H4, H8, H12, and H24 mean the data collected before and at 1, 2, 4, 8, 12, and 24 h HFNC, respectively. ROX, the ratio of SpO_2_/F_i_O_2_ to the respiratory rate; HFNC, high-flow nasal cannula; AUC, area under the curve of receiver operating characteristics; CI, confidence interval.

As the medical resources and staff were exhausted in the early stage of COVID-19, the data in ROX were missed in 5 patients before HFNC, 8 at 1, 24 at 2, 25 at 4, 25 at 8, 20 at 12, and 14 at 24 h. At 1, 2, 4, 8, 12, and 24 h and HFNC termination, the ROX index was much lower in patients experiencing HFNC failure than in those experiencing success (Figure 2A; Supplementary Table S2). In the multivariate analysis, we observed that the ROX index was independently associated with HFNC failure (odds ratio [OR] = 0.65; 95% confidence interval [CI]: 0.45–0.94) among the variables collected before and at 1 h of HFNC (Table 2). The AUC of the ROX index to predict HFNC failure was 0.74, 0.73, 0.73, 0.77, 0.75, and 0.79 at 1, 2, 4, 8, 12, and 24 h of HFNC, respectively (Figure 2B). Other variables to predict HFNC failure were summarized in Supplementary Table S3.

**TABLE 2:** Univariate and multivariate analysis for HFNC failure.

	Univariate analysis OR (95%CI)	*P* value	Multivariate analysis^a^ OR (95% CI)	*p* value
Age, years	1.05 (1.01–1.08)	0.02	−	−
SOFA score	1.39 (0.97–1.98)	0.07	2.16 (1.19–5.53)	0.02
Chronic respiratory disease	4.32 (1.03–18.12)	0.05	−	−
Systolic blood pressure before HFNC, mmHg	1.03 (1.00–1.06)	0.07	−	−
ROX index at 1 h of HFNC	0.68 (0.53–0.88)	<0.01	0.65 (0.45–0.94)	0.02

^a^ Due to missing data in some variables, 43 patients (22 HFNC successes and 21 failures) were entered in multivariate analysis.

HFNC, high-flow nasal cannula; OR, odds ratio; CI, confidence interval; SOFA, Sequential Organ Failure Assessment; ROX, the ratio of SpO_2_/F_i_O_2_ to the respiratory rate.

HFNC failure was defined as the requirement of escalation therapy (noninvasive ventilation or intubation).

The median duration of HFNC therapy was 242 h (IQR: 144–295) in the HFNC success group and 39 h (IQR: 15–117) in the group experiencing HFNC failure (Table 3). Among the patients with HFNC failure, six cases used NIV as a rescue therapy (21%), and 23 cases (79%) were directly intubated for invasive mechanical ventilation (IMV). Among the six NIV patients, two were intubated after NIV failure. Cardiac arrest occurred during HFNC therapy in four patients (6%), and all occurred in the resource-limited setting of Wuhan. Among the intubated patients, seven underwent extracorporeal membranous oxygenation (ECMO). The median time from HFNC initiation to intubation was 41 h (IQR: 19–152). Mortality was higher in patients with HFNC failure than in those with HFNC success (28 vs. 0%, *p* < 0.01).

**TABLE 3:** Outcomes of patients with HFNC success and failure.

	HFNC success (*N* = 37)	HFNC failure (*N* = 29)	*P* value
Duration of HFNC therapy, h	242 (144–295)	39 (15–117)	＜0.01
Duration of NIV, h	−	72 (21–192)	−
Duration of IMV, h	−	120 (48–576)	−
Length of ICU stay, d	16 (13–22)	15 (8–34)	0.92
Length of hospital stay, d	23 (17–33)	23 (8–42)	0.43
Cardiac arrest during HFNC, n %	−	4 (14)	−
NIV as a rescue therapy, n %	−	6 (21)	−
Intubation for IMV, n %	−	25 (86)	−
Time from initiation of HFNC to intubation, h	−	41 (19–152)	−
Use of ECMO, n %	−	7 (24)	−
Mortality, n %	0 (0)	14 (48)	＜0.01

HFNC, high-flow nasal cannula; NIV, noninvasive ventilation; IMV, invasive mechanical ventilation; ECMO, extracorporeal membranous oxygenation.

HFNC failure was defined as the requirement of escalation therapy (noninvasive ventilation or intubation).

### Comparisons Between Patients Inside and Outside Wuhan, China

**FIGURE 3:**
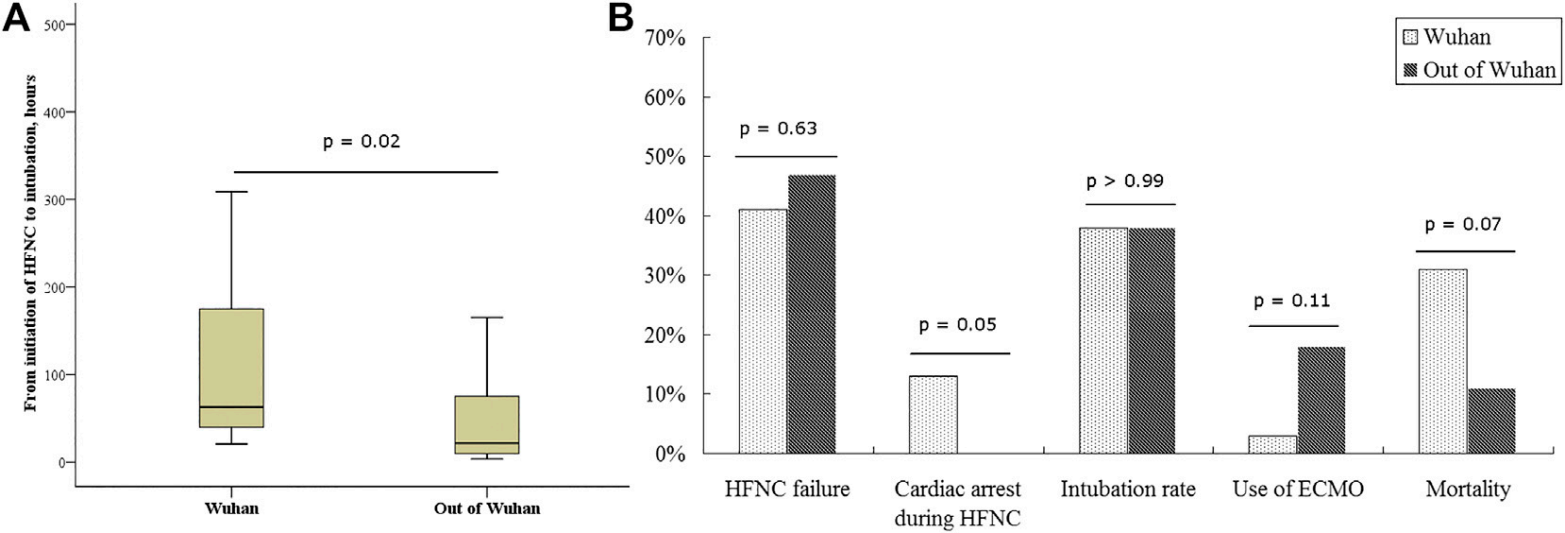
The comparisons between patients in and out of Wuhan. HFNC, high-flow nasal cannula; ECMO, extracorporeal membranous oxygenation. In Wuhan, a very large number of patients crowded into hospitals, staff were overwhelmed, and a severe shortage of medical devices occurred; thus, Wuhan is to be considered as a resource-limited area.

The rate of HFNC failure and intubation did not differ between patients inside and outside Wuhan (41 vs. 47% for HFNC failure, *p* = 0.63; 38 vs. 38% for intubation, *p* > 0.99). We also observed similar baseline characteristics of patients inside and outside Wuhan before HFNC initiation (Supplementary Table S4). However, the duration from HFNC initiation to intubation was longer in Wuhan than that outside Wuhan [63 (IQR: 39–179) vs. 22 (9–78) h, *p* = 0.02; Figure 3A]. Furthermore, all instances of cardiac arrests occurring under HFNC before intubation were in Wuhan, and all the cases died. Mortality trended higher in patients treated in Wuhan than in those treated outside Wuhan (31 vs. 12%, *p* = 0.07; Figure 3B).

## Discussion

In this study, we found the ROX index had a high predictive value to identify HFNC failure when it was measured within the first 24 h of HFNC therapy. Hospitals in Wuhan, as a resource-limited area, had similar HFNC failure rates but higher mortality than those centers outside Wuhan.

Several studies have reported the use of HFNC in COVID-19 patients (Geng et al., 2020; Wang et al., 2020; Chandel et al., 2020; Hu et al., 2020; Xu et al., 2020a). These studies showed that the rates of HFNC failure were between 38 and 45%, which agreed with the failure rate in our study (44%). We further explored if the resource limitation impacted the patients’ outcomes and found that resource limitation was associated with increased mortality. Although HFNC appeared feasible and successful in about half of the patients in a setting with adequate resources, monitoring of the ROX index may enable early identification of patients who are likely to require intubation; conversely, the use of HFNC in resource-limited settings without sufficient monitoring and delayed intubation may be associated with poor outcome, especially among those patients who were intubated at the last minute.

Prior to the COVID-19 pandemic, the ROX index showed high discriminative power to predict HFNC failure in hypoxemic patients (Roca et al., 2016). Recently, this team validated the ROX index in five ICUs in Spain and France (Roca et al., 2019). In our study, we have confirmed that the efficacy of the ROX index can be served as a predictor of HFNC failure among patients with COVID-19. The ROX index showed high discriminative values to predict HFNC failure within 24 h of HFNC use (at 1, 2, 4, 8, 12, and 24 h after HFNC initiation). As the variables required to calculate the ROX index are easy to obtain, even in the resource-limited area, it may be helpful for the physicians to early identify patients with a high likelihood of success and those who will require escalation therapy. Apart from the ROX index, the advanced age, increased SOFA score, and decreased platelets were also reported to be associated with increased HFNC failure (Hu et al., 2020; Xu et al., 2020a). As such, the combined use of these variables and the ROX index might help improve the predictive accuracy.

The surge of patients largely overloaded the healthcare workers and challenged medical resources (Cesari and Proietti, 2020; Solnica et al., 2020; Vergano et al., 2020). In China, most of the COVID-19 patients were in Wuhan. The duration from HFNC initiation to intubation for cases in Wuhan was longer than that outside Wuhan, and four cases with cardiac arrest during HFNC therapy all occurred in Wuhan. The present study did not record the reasons for this difference in duration from HFNC initiation to intubation, but it may indicate that delayed intubation occurred in Wuhan possibly because of lack of life-saving device resources or the overwhelmed staff. This might have contributed to the increased mortality observed in Wuhan compared with the mortality in patients outside Wuhan. Intensive monitoring during HFNC therapy is needed to avoid such delay in escalation therapy, such as awake prone positioning, NIV, or IMV (Tu et al., 2020; Xu et al., 2020b). As the ROX is easily obtained, it can be used to improve the management of COVID-19 patients in resource-limited circumstances to rapidly identify patients who will require escalation therapy, and thus, anticipate the required resources or plan the patient transfer.

Among the patients with COVID-19, Chandel et al. explored the likelihood of death in hospitals among patients with early and late HFNC failure (Chandel et al., 2020). Although the sample size was larger than that of ours, it failed to find the difference between patients with early and late HFNC failure. In Chandel et al.’s study, the patients who required intubation within 48 h of HFNC were classified as early HFNC failure, in contrast to the late HFNC failure in which patients were intubated after 48 h of HFNC. This definition is unable to distinguish the duration of the hypoxemia, especially severe hypoxemia. A longer duration of hypoxemia was more likely to be associated with higher mortality. In our study, we classified the patients with and without resource limitation. The patients in resource-limited areas were bound to experience a longer duration of hypoxemia and delayed escalation care, which might explain the higher mortality in the resource-limited area.

This study has several limitations. First, only 43 patients (65%) were included in the multivariate analysis as some variables were missing due to retrospective design. And data imputation was not deemed feasible, given the small sample size. Consequently, it was not possible to combine several variables to predict HFNC failure with greater accuracy. Second, even though all the centers had built HFNC protocol and keeping SpO_2_ above 93% was the goal, it is impossible to guarantee that the goal would be achieved all the time for all the patients, as the data points were not recorded minute by minute in the medical records. Third, delayed therapy may occur due to the bedside treating physician. Fourth, a lack of power may have resulted in the lack of statistically significant mortality between the patients with delayed intubation in Wuhan compared to those promptly intubated in the absence of resource limitation outside Wuhan. Last, HFNC gas flow settings were found to affect the ROX index (Mauri et al., 2019), due to room air entrainment when the gas flow is set below the patient inspiratory flow demand. As such, a constant gas flow setting might enable a more precise ROX index measurement but might not be feasible in a clinical study.

## Conclusion

ROX index, calculated by the ratio of SpO_2_/FiO_2_ to the respiratory rate, is easily obtained at bedside and can be used to predict HFNC failure among the patients with COVID-19. It may be used to avoid delayed escalation care, which may otherwise occur in resource-limited areas.

## Data Availability

The raw data supporting the conclusions of this article will be made available by the authors, without undue reservation.

## References

[B1] Alhazzani W. Møller M. H. Arabi Y. M. Loeb M. Gong M. N. Fan E. (2020). Surviving sepsis campaign: guidelines on the management of critically ill adults with coronavirus disease 2019 (COVID-19). Intensive Care Med. 46, 854–887. 10.1007/s00134-020-06022-5 32222812 PMC7101866

[B2] Bhatraju P. K. Ghassemieh B. J. Nichols M. Kim R. Jerome K. R. Nalla A. K. (2020). Covid-19 in critically ill patients in the seattle region–case series. N. Engl. J. Med. 382, 2012. 10.1056/NEJMoa2004500 32227758 PMC7143164

[B3] Cesari M. Proietti M. (2020). COVID-19 in Italy: ageism and decision making in a pandemic. J. Am. Med. Dir. Assoc. 21, 576–577. 10.1016/j.jamda.2020.03.025 32334771 PMC7118618

[B4] Chandel A. Patolia S. Brown A. W. Collins A. C. Sahjwani D. Khangoora V. (2020). High-flow nasal cannula in COVID-19: outcomes of application and examination of the ROX index to predict success. Respir. Care 66, respcare.08631. 10.4187/respcare.08631 33328179

[B5] Critical care committee of Chinese Association of Chest Physician (2020). [Conventional respiratory support therapy for severe acute respiratory infections (SARI): clinical indications and nosocomial infection prevention and control]. Zhonghua Jie He He Hu Xi Za Zhi 43, 189–194. 10.3760/cma.j.issn.1001-0939.2020.03.010 32164086

[B6] Gattinoni L. Coppola S. Cressoni M. Busana M. Rossi S. Chiumello D. (2020a). Covid-19 does not lead to a “typical” acute respiratory distress syndrome. Am. J. Respir. Crit. Care Med. 201, 1299. 10.1164/rccm.202003-0817LE 32228035 PMC7233352

[B7] Gattinoni L. Chiumello D. Caironi P. Busana M. Romitti F. Brazzi L. (2020b). COVID-19 pneumonia: different respiratory treatments for different phenotypes?. Intensive Care Med. 46, 1099. 10.1007/s00134-020-06033-2 32291463 PMC7154064

[B8] Geng S. Mei Q. Zhu C. Yang T. Yang Y. Fang X. (2020). High flow nasal cannula is a good treatment option for COVID-19. Heart Lung 49 (5), 444–445. 10.1016/j.hrtlng.2020.03.018 32295710 PMC7151489

[B9] Hu M. Zhou Q. Zheng R. Li X. Ling J. Chen Y. (2020). Application of high-flow nasal cannula in hypoxemic patients with COVID-19: a retrospective cohort study. BMC Pulm. Med. 20, 324. 10.1186/s12890-020-01354-w 33357219 PMC7758183

[B10] Hui D. S. Chow B. K. Lo T. Tsang O. T. Y. Ko F. W. Ng S. S. (2019). Exhaled air dispersion during high-flow nasal cannula therapy versus CPAP via different masks. Eur. Respir. J. 53. 10.1183/13993003.02339-2018 30705129

[B11] Kang B. J. Koh Y. Lim C. M. Huh J. W. Baek S. Han M. (2015). Failure of high-flow nasal cannula therapy may delay intubation and increase mortality. Intensive Care Med. 41, 623–632. 10.1007/s00134-015-3693-5 25691263

[B12] Li J. Fink J. B. Ehrmann S. (2020a). High-flow nasal cannula for COVID-19 patients: low risk of bio-aerosol dispersion. Eur. Respir. J. 55, 2000892. 10.1183/13993003.00892-2020 32299867 PMC7163690

[B13] Li J. Jing G. Scott J. B. (2020b). Year in review 2019: high-flow nasal cannula oxygen therapy for adult subjects. Respir. Care 65, 545–557. 10.4187/respcare.07663 32213602

[B14] Mauri T. Carlesso E. Spinelli E. Turrini C. Corte F. D. Russo R. (2019). Increasing support by nasal high flow acutely modifies the rox index in hypoxemic patients: a physiologic study. J. Crit. Care 53, 183–185. 10.1016/j.jcrc.2019.06.020 31254849

[B15] Nishimura M. (2016). High-flow nasal cannula oxygen therapy in adults: physiological benefits, indication, clinical benefits, and adverse effects. Respir. Care 61, 529–541. 10.4187/respcare.04577 27016353

[B16] Respiratory and Critical Care Medicine Group of Chinese Thoracic Society (2019). [Expert consensus of high flow nasal cannula oxygen therapy on clinical application regularity]. Zhonghua Jie He He Hu Xi Za Zhi 42, 83–91. 10.3760/cma.j.issn.1001-0939.2019.02.003 30704179

[B17] Roca O. Caralt B. Messika J. Samper M. Sztrymf B. Hernández G. (2019). An index combining respiratory rate and oxygenation to predict outcome of nasal high-flow therapy. Am. J. Respir. Crit. Care Med. 199, 1368–1376. 10.1164/rccm.201803-0589OC 30576221

[B18] Roca O. Messika J. Caralt B. García-de-Acilu M. Sztrymf B. Ricard J. D. (2016). Predicting success of high-flow nasal cannula in pneumonia patients with hypoxemic respiratory failure: the utility of the ROX index. J. Crit. Care 35, 200–205. 10.1016/j.jcrc.2016.05.022 27481760

[B19] Solnica A. Barski L. Jotkowitz A. (2020). Allocation of scarce resources during the COVID-19 pandemic: a Jewish ethical perspective. J. Med. Ethics 46, 444. 10.1136/medethics-2020-106242 32277021

[B20] Sun Q. Qiu H. Huang M. Yang Y. (2020). Lower mortality of COVID-19 by early recognition and intervention: experience from Jiangsu Province. Ann. Intensive Care 10, 33. 10.1186/s13613-020-00650-2 32189136 PMC7080931

[B21] Tu G. W. Liao Y. X. Li Q. Y. Dong H. Yang L. Y. Zhang X. Y. (2020). Prone positioning in high-flow nasal cannula for COVID-19 patients with severe hypoxemia: a pilot study. Ann. Transl. Med. 8, 598. 10.21037/atm-20-3005 32566624 PMC7290555

[B22] Vergano M. Bertolini G. Giannini A. Gristina G. R. Livigni S. Mistraletti G. (2020). Clinical ethics recommendations for the allocation of intensive care treatments in exceptional, resource-limited circumstances: the Italian perspective during the COVID-19 epidemic. Crit. Care 24, 165. 10.1186/s13054-020-02891-w 32321562 PMC7175451

[B23] Wang K. Zhao W. Li J. Shu W. Duan J. (2020). The experience of high-flow nasal cannula in hospitalized patients with 2019 novel coronavirus-infected pneumonia in two hospitals of Chongqing, China. Ann. Intensive Care 10, 37. 10.1186/s13613-020-00653-z 32232685 PMC7104710

[B24] WHO Coronavirus Disease Dashboard (2021). WHO Coronavirus Disease Dashboard. Available at: https://arcg.is/XvuSX (Accessed January 17, 2021).

[B25] Wu Z. McGoogan J. M. (2020). Characteristics of and important lessons from the coronavirus disease 2019 (COVID-19) outbreak in China. JAMA 323, 1239. 10.1001/jama.2020.2648 32091533

[B26] Xu J. Yang X. Huang C. Zou X. Zhou T. Pan S. (2020a). A novel risk-stratification models of the high-flow nasal cannula therapy in COVID-19 patients with hypoxemic respiratory failure. Front. Med. 7, 607821. 10.3389/fmed.2020.607821 PMC7793962 33425951

[B27] Xu Q. Wang T. Qin X. Jie Y. Zha L. Lu W. (2020b). Early awake prone position combined with high-flow nasal oxygen therapy in severe COVID-19: a case series. Crit. Care 24, 250. 10.1186/s13054-020-02991-7 32448330 PMC7246000

[B28] Yang X. Yu Y. Xu J. Shu H. Xia J. Liu H. (2020). Clinical course and outcomes of critically ill patients with SARS-CoV-2 pneumonia in Wuhan, China: a single-centered, retrospective, observational study. Lancet Respir. Med. 8 (5), 475–481. 10.1016/S2213-2600(20)30079-5 32105632 PMC7102538

[B29] Youden W. J. (1950). Index for rating diagnostic tests. Cancer 3 (1), 32–35. 10.1002/1097-0142(1950)3:1<32::aid-cncr2820030106>3.0.co;2-3 15405679

[B30] Yuan X. Mu J. S. Mo G. X. Hu X. S. Yan P. Xie L. X. (2020). [Respiratory support for severe 2019-nCoV pneumonia suffering from acute respiratory failure: time and strategy]. Zhonghua Jie He He Hu Xi Za Zhi 43, 177–180. 10.3760/cma.j.issn.1001-0939.2020.03.006 32164082

[B31] Ziehr D. R. Alladina J. Petri C. R. Maley J. H. Moskowitz A. Medoff B. D. (2020). Respiratory pathophysiology of mechanically ventilated patients with COVID-19: a cohort study. Am. J. Respir. Crit. Care. Med. 201, 1560. 10.1164/rccm.202004-1163LE 32348678 PMC7301734

