# Supplementary material for: High-Flow Nasal Cannula for COVID-19 Patients: A Multicenter Retrospective Study in China
Source: Front Mol Biosci. 2021 Apr 13;8:639100. doi: 10.3389/fmolb.2021.639100 (PMC8078589; doi:10.3389/fmolb.2021.639100)
Supplement: Supplementary file 1 [file table1.doc]

Supplementary table 1. Baseline data collected at hospital admission in patients with HFNC success and failure

|  | HFNC success  N = 37 | HFNC failure  N = 29 | *p* |
| --- | --- | --- | --- |
| Airway secretions |  |  |  |
| None | 23 (62%) | 14 (48%) | 0.32 |
| Mild | 13 (35%) | 15 (52%) | 0.21 |
| Moderate to abundant | 1 (3%) | 0 (0%) | ＞0.99 |
| Laboratory tests |  |  |  |
| White blood cell counts, ×10^9^/L | 7.2 ± 3.9 | 8.7 ± 5.1 | 0.19 |
| Lymphocyte counts, ×10^9^/L | 0.97 ± 0.89 | 0.87 ± 0.56 | 0.60 |
| PCT, ng/mL | 0.08 (0.05-0.10) | 0.21 (0.07-1.67) | ＜0.01 |
| IL-6 | 18 (2-54) | 17 (7-77) | 0.61 |
| C-reactive protein, mg/L | 68 ± 63 | 89 ± 77 | 0.27 |
| LDH, U/L | 339 ± 86 | 363 ± 136 | 0.53 |
| CD4, counts/μL | 312 ± 168 | 215 ± 184 | 0.21 |
| pH | 7.42 ± 0.05 | 7.42 ± 0.08 | 0.78 |
| PaCO_2_, mmHg | 40 ± 9 | 39 ± 10 | 0.60 |
| PaO_2_/F_I_O_2_, mmHg | 264 ± 137 | 279 ± 123 | 0.70 |
| Lactate, mmol/L | 2.3 ± 0.9 | 2.8 ± 1.4 | 0.13 |
| Vital signs |  |  |  |
| Heart rate, beats/min | 87 ± 13 | 94 ± 15 | 0.07 |
| Respiratory rate, breaths/min | 22 ± 4 | 23 ± 5 | 0.22 |
| Systolic blood pressure, mmHg | 125 ± 21 | 136 ± 20 | 0.03 |
| Diastolic blood pressure, mmHg | 72 ± 9 | 75 ± 13 | 0.27 |
| SpO_2_, % | 96 (92-98) | 95 (92-98) | 0.94 |
| ROX index | 12.1 ± 5.3 | 11.9 ± 5.7 | 0.88 |

HFNC = high-flow nasal cannula, PCT = procalcitonin, LDH = lactate dehydrogenase, ROX = the ratio of SpO_2_/F_I_O_2_ to respiratory rate

HFNC failure was defined as requirement of escalation therapy (noninvasive ventilation or intubation).
