# Supplementary material for: High-Flow Nasal Cannula for COVID-19 Patients: A Multicenter Retrospective Study in China
Source: Front Mol Biosci. 2021 Apr 13;8:639100. doi: 10.3389/fmolb.2021.639100 (PMC8078589; doi:10.3389/fmolb.2021.639100)
Supplement: Supplementary file 2 [file table2.doc]

Supplementary Table 2. Data collected from 1 h to termination of HFNC

|  | HFNC success  N = 37 | HFNC failure  N = 29 | *p* |
| --- | --- | --- | --- |
| Variables collected at 1 h of HFNC |  |  |  |
| pH | 7.41 ± 0.05 | 7.42 ± 0.04 | 0.54 |
| PaCO_2_, mmHg | 41 ± 9 | 41 ± 9 | 0.78 |
| PaO_2_/F_I_O_2_, mmHg | 189 ± 90 | 130 ± 86 | 0. 06 |
| Lactate, mmol/L | 2.4 ± 1.2 | 2.0 ± 0.9 | 0.38 |
| Heart rate, beats/min | 88 ± 10 | 91 ± 16 | 0.34 |
| Respiratory rate, breaths/min | 22 ± 4 | 25 ± 6 | 0.03 |
| Systolic blood pressure, mmHg | 124 ± 16 | 125 ± 18 | 0.73 |
| Diastolic blood pressure, mmHg | 74 ± 8 | 70 ± 9 | 0.13 |
| SpO_2_, % | 96 (93-98) | 93 (88-96) | ＜0.01 |
| ROX index | 8.2 ± 2.2 | 6.2 ± 4.5 | ＜0.01 |
| Flow, L/min | 40 ± 10 | 44 ± 12 | 0.14 |
| Variables collected at 2 h of HFNC |  |  |  |
| pH | 7.41 ± 0.04 | 7.42 ± 0.07 | 0.60 |
| PaCO_2_, mmHg | 40 ± 10 | 39 ± 9 | 0.83 |
| PaO_2_/F_I_O_2_, mmHg | 197 ± 74 | 110 ± 70 | 0.03 |
| Lactate, mmol/L | 2.7 ± 1.2 | 2.1 ± 0.8 | 0.29 |
| Heart rate, beats/min | 88 ± 13 | 87 ± 17 | 0.88 |
| Respiratory rate, breaths/min | 21 ± 4 | 25 ± 8 | 0.08 |
| Systolic blood pressure, mmHg | 126 ± 13 | 126 ± 14 | 0.98 |
| Diastolic blood pressure, mmHg | 73 ± 8 | 74 ± 8 | 0.75 |
| SpO_2_, % | 96 (95-98) | 94 (89-96) | ＜0.01 |
| ROX index | 9.2 ± 2.6 | 7.0 ± 4.1 | 0.04 |
| Flow, L/min | 37 ± 10 | 46 ± 14 | 0.01 |
| Variables collected at 4 h of HFNC |  |  |  |
| pH | 7.40 ± 0.04 | 7.40 ± 0.07 | 0.88 |
| PaCO_2_, mmHg | 42 ± 8 | 43 ± 6 | 0.68 |
| PaO_2_/F_I_O_2_, mmHg | 212 ± 86 | 101 ± 18 | ＜0.01 |
| Lactate, mmol/L | 2.3 ± 1.1 | 2.1 ± 0.7 | 0.80 |
| Heart rate, beats/min | 84 ± 11 | 86 ± 19 | 0.69 |
| Respiratory rate, breaths/min | 21 ± 3 | 23 ± 6 | 0.10 |
| Systolic blood pressure, mmHg | 128 ± 11 | 122 ± 14 | 0.14 |
| Diastolic blood pressure, mmHg | 71 ± 9 | 71 ± 7 | 0.98 |
| SpO_2_, % | 97 (95-98) | 94 (90-96) | ＜0.01 |
| ROX index | 9.4 ± 2.8 | 7.0 ± 3.1 | 0.02 |
| Flow, L/min | 35 ± 9 | 43 ± 13 | 0.03 |
| Variables collected at 8 h of HFNC |  |  |  |
| pH | 7.44 ± 0.04 | 7.36 ± 0.10 | 0.07 |
| PaCO_2_, mmHg | 39 ± 5 | 47 ± 10 | 0.07 |
| PaO_2_/F_I_O_2_, mmHg | 214 ± 92 | 81 ± 10 | 0. 02 |
| Lactate, mmol/L | 2.3 ± 0.8 | 1.6 ± 0.6 | 0.16 |
| Heart rate, beats/min | 85 ± 17 | 92 ± 15 | 0.21 |
| Respiratory rate, breaths/min | 21 ± 4 | 25 ± 8 | 0.03 |
| Systolic blood pressure, mmHg | 128 ± 17 | 128 ± 14 | 0.96 |
| Diastolic blood pressure, mmHg | 71 ± 7 | 76 ± 10 | 0.08 |
| SpO_2_, % | 97 (96-98) | 93 (90-96) | ＜0.01 |
| ROX index | 9.2 ± 2.7 | 6.6 ± 3.1 | ＜0.01 |
| Flow, L/min | 36 ± 10 | 42 ± 12 | 0.07 |
| Variables collected at 12 h of HFNC |  |  |  |
| pH | 7.43 ± 0.04 | 7.42 ± 0.11 | 0.66 |
| PaCO_2_, mmHg | 37 ± 6 | 44 ± 9 | 0.04 |
| PaO_2_/F_I_O_2_, mmHg | 216 ± 89 | 120 ± 63 | 0.02 |
| Lactate, mmol/L | 2.3 ± 1.0 | 2.0 ± 0.9 | 0.50 |
| Heart rate, beats/min | 86 ± 14 | 90 ± 17 | 0.37 |
| Respiratory rate, breaths/min | 21 ± 4 | 25 ± 8 | 0.07 |
| Systolic blood pressure, mmHg | 125 ± 12 | 128 ± 16 | 0.47 |
| Diastolic blood pressure, mmHg | 72 ± 7 | 74 ± 12 | 0.35 |
| SpO_2_, % | 98 (96-99) | 94 (90-96) | ＜0.01 |
| ROX index | 9.0 ± 2.7 | 6.9 ± 3.8 | 0.03 |
| Flow, L/min | 37 ± 9 | 42 ± 12 | 0.09 |
| Variables collected at 24 h of HFNC |  |  |  |
| pH | 7.44 ± 0.04 | 7.43 ± 0.09 | 0.74 |
| PaCO_2_, mmHg | 39 ± 6 | 38 ± 8 | 0.68 |
| PaO_2_/F_I_O_2_, mmHg | 216 ± 148 | 129 ± 86 | 0.05 |
| Lactate, mmol/L | 2.2 ± 0.9 | 2.5 ± 1.4 | 0.44 |
| Heart rate, beats/min | 80 ± 10 | 90 ± 17 | ＜0.01 |
| Respiratory rate, breaths/min | 22 ± 4 | 25 ± 5 | 0.02 |
| Systolic blood pressure, mmHg | 125 ± 15 | 124 ± 18 | 0.85 |
| Diastolic blood pressure, mmHg | 72 ± 7 | 71 ± 13 | 0.57 |
| SpO_2_, % | 98 (96-99) | 95 (90-98) | ＜0.01 |
| ROX index | 9.0 ± 3.1 | 6.4 ± 2.6 | ＜0.01 |
| Flow, L/min | 40 ± 10 | 42 ± 11 | 0.45 |
| White blood cell counts, ×10^9^/L | 9.7 ± 6.8 | 11.2 ± 3.8 | 0.38 |
| Lymphocyte count, ×10^9^/L | 0.95 ± 0.56 | 0.65 ± 0.42 | 0.03 |
| PCT, ng/mL | 0.07 (0.04-0.10) | 0.31 (0.18-0.73) | ＜0.01 |
| IL-6 | 40 (3-534) | 62 (9-104) | 0.42 |
| C-reactive protein, mg/L | 51 ± 41 | 100 ± 63 | ＜0.01 |
| LDH, U/L | 382 ± 105 | 486 ± 226 | 0.11 |
| CD4, counts/μL | 230 ± 109 | 180 ± 27 | 0.48 |
| Variables collected at termination of HFNC |  |  |  |
| pH | 7.40 ± 0.04 | 7.37 ± 0.15 | 0.34 |
| PaCO_2_, mmHg | 43 ± 7 | 50 ± 22 | 0.17 |
| PaO_2_/F_I_O_2_, mmHg | 250 ± 87 | 107 ± 75 | ＜0.01 |
| Lactate, mmol/L | 2.1 (1.6-2.4) | 2.1 (1.5-4.3) | 0.61 |
| Heart rate, beats/min | 84 ± 12 | 94 ± 28 | 0.07 |
| Respiratory rate, breaths/min | 19 ± 5 | 26 ± 9 | ＜0.01 |
| Systolic blood pressure, mmHg | 124 ± 15 | 115 ± 27 | 0.14 |
| Diastolic blood pressure, mmHg | 69 ± 9 | 64 ± 16 | 0.31 |
| SpO_2_, % | 98 (97-99) | 89 (84-91) | ＜0.01 |
| ROX index | 15.2 ± 6.1 | 5.1 ± 2.5 | ＜0.01 |
| Flow, L/min | 31 ± 8 | 48 ± 13 | ＜0.01 |
| White blood cell counts, ×10^9^/L | 8.5 ± 6.2 | 11.6 ± 4.1 | 0.04 |
| Lymphocyte counts, ×10^9^/L | 1.26 ± 0.53 | 0.67 ± 0.47 | ＜0.01 |
| PCT, ng/mL | 0.04 (0.03-0.10) | 0.49 (0.17-0.90) | ＜0.01 |
| IL-6 | 7 (1-23) | 67 (7-95) | 0.03 |
| C-reactive protein, mg/L | 9 (3-21) | 88 (40-142) | ＜0.01 |
| LDH, U/L | 256 ± 85 | 462 ± 244 | ＜0.01 |
| CD4, counts/μL | 601 ± 359 | 193 ± 106 | 0.03 |

HFNC = high-flow nasal cannula, PCT = procalcitonin, LDH = lactate dehydrogenase, ROX = the ratio of SpO_2_/F_I_O_2_ to respiratory rate

HFNC failure was defined as requirement of escalation therapy (noninvasive ventilation or intubation).
