# Supplementary material for: High-Flow Nasal Cannula for COVID-19 Patients: A Multicenter Retrospective Study in China
Source: Front Mol Biosci. 2021 Apr 13;8:639100. doi: 10.3389/fmolb.2021.639100 (PMC8078589; doi:10.3389/fmolb.2021.639100)
Supplement: Supplementary file 3 [file table3.doc]

Supplementary table 3. Prediction of HFNC failure tested by selected variables

|  | AUC (95%CI) | Cutoff value | Sensitivity, % | Specificity,% |
| --- | --- | --- | --- | --- |
| Age, years | 0.68 (0.55-0.79) | ＞76 | 55% | 78% |
| SOFA before HFNC | 0.73 (0.59-0.84) | ＞3 | 76% | 61% |
| PCT at admission, ng/mL | 0.71 (0.57-0.83) | ＞0.15 | 58% | 86% |
| PCT before HFNC, ng/mL | 0.75 (0.59-0.87) | ＞0.16 | 69% | 88% |
| Lymphocyte counts before HFNC, ×10^9^/L | 0.83 (0.70-0.92) | ≤0.73 | 82% | 74% |
| PaO_2_/F_I_O_2_ at 1 h of HFNC, mmHg | 0.79 (0.62-0.91) | ≤107 | 63% | 100% |

HFNC = high-flow nasal cannula, AUC = area under the curve of receiver operating characteristics, CI = confidence interval, SOFA = sequential organ failure assessment, PCT = procalcitonin

HFNC failure was defined as requirement of escalation therapy (noninvasive ventilation or intubation).
