# Supplementary material for: High-Flow Nasal Cannula for COVID-19 Patients: A Multicenter Retrospective Study in China
Source: Front Mol Biosci. 2021 Apr 13;8:639100. doi: 10.3389/fmolb.2021.639100 (PMC8078589; doi:10.3389/fmolb.2021.639100)
Supplement: Supplementary file 4 [file table4.doc]

Supplementary table 4. Comparisons between patients in and out of Wuhan

|  | Wuhan  N = 32 | Out of Wuhan  N = 34 | *p* |
| --- | --- | --- | --- |
| Age, years | 72 ± 16 | 63 ± 16 | 0.03 |
| Male (%) | 14 (44%) | 11 (32%) | 0.45 |
| Oxygen therapy before HFNC, % | 2 (6%) | 10 (29%) | 0.02 |
| Airway secretions |  |  |  |
| None | 10 (31%) | 22 (65%) | <0.01 |
| Mild | 21 (66%) | 12 (35%) | 0.03 |
| Moderate to abundant | 1 (3%) | 0 (0%) | 0.49 |
| Underlying disease |  |  |  |
| Hypertension | 21 (67%) | 19 (56%) | 0.46 |
| Diabetes mellitus | 8 (25%) | 11 (32%) | 0.59 |
| Coronary heart disease | 6(19%) | 2 (6%) | 0.14 |
| Cerebral infarction | 6(19%) | 2 (6%) | 0.14 |
| Chronic respiratory disease | 8 (25%) | 3 (9%) | 0.10 |
| Hypoproteinemia | 13 (41%) | 0 (0%) | <0.01 |
| Anemia | 9 (28%) | 0 (0%) | <0.01 |
| Chronic renal dysfunction | 4 (13%) | 1 (3%) | 0.19 |
| Gastrointestinal bleeding | 1 (3%) | 3 (9%) | 0.61 |
| Variables collected before HFNC |  |  |  |
| SOFA score | 3.5 ± 1.8 | 4.2 ± 2.1 | 0.22 |
| White blood cell counts, ×10^9^/L | 9.8 ± 4.3 | 7.6 ± 3.7 | 0.06 |
| Lymphocyte counts, ×10^9^/L | 0.79 (0.61-1.21) | 0.69 (0.51-0.85) | 0.15 |
| PCT, ng/mL | 0.10 (0.06-2.9) | 0.10 (0.05-0.42) | 0.69 |
| IL-6 | 57 (8-76) | 16 (0.3-192) | 0.42 |
| C-reactive protein, mg/L | 74 ± 70 | 83 ± 55 | 0.62 |
| LDH, U/L | 340 ± 108 | 409 ± 135 | 0.25 |
| pH | 7.43 ± 0.09 | 7.42 ± 0.05 | 0.70 |
| PaCO_2_, mmHg | 41 (35-51) | 36 (32-42) | 0.09 |
| PaO_2_/F_I_O_2_, mmHg | 169 ± 102 | 210 ± 115 | 0.22 |
| Lactate, mmol/L | 3.0 ± 1.5 | 2.5 ± 1.3 | 0.21 |
| Heart rate, beats/min | 89 ± 15 | 94 ± 17 | 0.19 |
| Respiratory rate, breath/min | 24 ± 5 | 26 ± 6 | 0.05 |
| Systolic blood pressure, mmHg | 132 ± 21 | 120 ± 17 | 0.02 |
| Diastolic blood pressure, mmHg | 73 ± 9 | 69 ± 9 | 0.10 |
| SpO_2_, % | 92 (86-96) | 93 (89-95) | 0.82 |
| ROX index | 8.7 ± 2.8 | 9.2 ± 4.9 | 0.59 |
| Outcomes |  |  |  |
| Duration of HFNC, h | 175 (43-285) | 138 (22-218) | 0.07 |
| Duration of IMV, h | 24 (12-72) | 145 (92-720) | 0.04 |
| The length of ICU stay, d | 13 (7-19) | 21 (15-35) | <0.01 |
| The length of hospital stay, d | 15 (7-30) | 26 (22-44) | <0.01 |
| HFNC failure, % | 13 (41%) | 16 (47%) | 0.63 |
| Cardiac arrest during HFNC, % | 4 (13%) | 0 (0%) | 0.05 |
| NIV as a rescue therapy, % | 2 (6%) | 4 (12%) | 0.67 |
| Intubation for IMV, % | 12 (38%) | 13 (38%) | >0.99 |
| Time from HFNC initiation to intubation, h | 63 (39-179) | 22 (9-78) | 0.02 |
| Use of ECMO, % | 1 (3%) | 6 (18%) | 0.11 |
| Mortality, % | 10 (31%) | 4 (12%) | 0.07 |

PCT = procalcitonin, LDH = lactate dehydrogenase, ROX = the ratio of SpO_2_/F_I_O_2_ to respiratory rate, HFNC = high-flow nasal cannula, SOFA = sequential organ failure assessment, NIV = noninvasive ventilation, IMV = invasive mechanical ventilation, ECMO = extracorporeal membranous oxygenation
